# Reproductive Justice for Adolescent Migrant Girls in the Post-ICPD Era: A Critical Analysis of Contraceptive Care for Venezuelan Girls in Colombia

**DOI:** 10.1007/s12116-025-09474-2

**Published:** 2025-07-07

**Authors:** Hannah Louise Hall

**Affiliations:** Department of Politics and International Relations, University of Southampton, Southampton, UK

**Keywords:** Sexual and reproductive health, Sexual and reproductive health rights, Reproductive justice, Adolescent sexual and reproductive health, Migration, Gender and development, Humanitarianism, Contraceptive care

## Abstract

Adolescent migrant girls face unique challenges to their sexual and reproductive health and rights (SRHR) due to their intersecting identities as women, young people, and migrants. Since the first International Conference on Population and Development (ICPD) in 1994, reports have increasingly emphasized the prioritization of SRHR for marginalized groups. In this article, I examine the response to contraceptive care needs through the case study of Venezuelan adolescent migrant girls in Colombia, utilizing Reproductive Justice as an analytical framework. I evaluate how state and non-state actors’ responses address the intersections of gender, age, and migration to create environments that discourage contraceptive care access. I collected qualitative data through multi-perspective interviews conducted during ethnographic fieldwork in 2022. The sample includes 30 adolescent migrant girls and key informants involved in designing and delivering policies or programs for Venezuelan migrants in Colombia. Overall, the findings show that both state and non-state actors favored short-term, “urgent” interventions over access to adolescent-friendly, migrant-inclusive contraceptive care. I conclude that responses fragment aspects of SRH, deprioritizing contraceptive care and other longer-term intersectional responses in favor of urgent responses, which, in turn, further marginalize adolescent migrant girls. To overcome this, the post-ICPD agenda must recognize SRHR as a continuum and shape responses, aligning responses with the reproductive realities of adolescent migrant girls.

## Introduction

The International Conference on Population and Development (ICPD), Cairo, 1994, was a landmark event in the history of sexual and reproductive health and rights (SRHR). Its Program of Action affirmed that all individuals have the right to make informed decisions about their bodies and to access safe and effective reproductive health care services (UNFPA 2014). Moreover, the ICPD Program of Action encouraged states to address situations of vulnerability that may curtail the abilities of certain groups to enjoy SRHR.^1^

Thirty years later, I argue that state-led and humanitarian responses fragment aspects of sexual and reproductive health (SRH) deprioritizing contraceptive care and other longer-term intersectional responses in favor of “urgent” responses that provide basic emergency care to the migrant population. As a result, shorter-term emergency responses fail to address the reproductive realities in which adolescent migrant girls seek contraceptive care, creating an environment where access to contraceptive care is made more difficult depending on age, gender, and migration status. This can be seen in the current attempts to respond to the contraceptive needs of Venezuelan adolescent migrant girls. While these responses may be imbued with the spirit of the ICPD, in practice, they run the risk of perpetuating reproductive injustices.

Driven by a desire to understand the ways state and non-state actors address adolescent migrant girls’ reproductive realities, my analysis begins with the international obligations made to adolescent migrant girls in the ICPD and subsequent national-level policies.^2^ Then, I discuss whether policies and programs regarding contraceptive care are implemented in accordance with these commitments and the broader framework of Reproductive Justice (RJ).

In this article, I draw on the case study of Venezuelan migrants in Colombia. The socioeconomic crisis in Venezuela has led to a large number of adolescent girls migrating to Colombia. In 2015, there were 48,714 Venezuelan migrants in Colombia; as of 2022, this number rose to 2,477,588, with many entering irregularly (R4 V 2023). Estimates show that young women and girls aged 5–29 make up 27.24% of the Venezuelans who migrate to Colombia (Migración Colombia 2023).

Importantly, poor maternal and neonatal health outcomes are key issues facing a large proportion of Venezuelan migrants (Doocy et al. 2019). In their country of origin, many Venezuelans experience malnutrition, lack of resources and services, and heightened levels of disease (Ibid.), culminating in increased indicators of maternal, neonatal, and child mortality. Between 2015 and 2016, Venezuela experienced a 66% increase in maternal mortality and a 30% rise in infant mortality (Fraser, 2017). The lack of SRH care extends to contraceptive care. In 2018, it was estimated that 80% of women had an unmet need for contraception (UNFPA 2019b). Reports suggest that many contraceptive methods are only available at 25 times their price on the black market (Albaladejo, 2018; CARE 2019). The rise in unintended pregnancies in Venezuela, where abortion is both illegal and subject to strong social stigma, has led many women to seek unsafe abortions (Profamilia 2019). Since 2015, an unmet need for contraceptives in Venezuela has been linked to a 65% increase in adolescent pregnancy (ACNUDH 2019). Consequently, Venezuelan migrant women and girls name hunger, poverty, violence, a need for medical attention, and family reunion as the main drivers of displacement (Riggirozzi et al. 2023, 3757).

Considering the gaps in care experienced by Venezuelan migrant women and girls, I seek to understand how state actors (e.g., government ministries, public health authorities) and non-state actors (international and national NGOs, private healthcare providers, multilateral agencies, and civil society actors) have responded to meet contraceptive needs.

I agree with Lafrenière, Sweetman, and Thylin (2019) and Foley and Hendrixson (2011), who argue that responses framed in crisis terms discount gendered forms of oppression. Migration is a gendered phenomenon that leads to specific injustices for women and girls (Piper 2018). Venezuelan women and girls often migrate to Colombia to seek SRH care in addition to food and employment (Doocy et al. 2019; Cintra, Owen, and Riggirozzi 2023). Along this journey, Venezuelan women and girls are at a heightened risk of violence, forced marriage, trafficking, and exploitation (Doocy et al. 2019, 82; Calderon-Jaramillo et al. 2020). This indicates a need for responses that match the gendered forms of reproductive injustices that occur during migration.

Given this, I argue that gender alone is an insufficient lens through which to view SRH in migration in humanitarian crises. Age must also be considered.

Most studies in Colombia have focused on women aged 15–49, opting to fold all women of reproductive age into one large group. This assumes a level of shared experiences for all women based on assumed reproductive capability, even though most 15-year-olds’ life experiences are different from a 30-year-old’s (Flórez García et al. 2020; Doocy et al. 2019; Calderon-Jaramillo et al. 2020; Murillo-Pedrozo et al. 2021). For example, studies that use disaggregated data have found that younger people experience greater exposure to violence and difficulties accessing services than their counterparts in their 20s (Profamilia 2019; Ortiz-Ruiz et al. 2023). Migrants state that contraceptive methods and adolescent-friendly services are some of the most prevalent unmet needs (Profamilia 2019). Yet the roles and responsibilities of host governments and humanitarian actors in meeting the contraceptive care needs of adolescent migrant girls are not well understood. Accordingly, I argue that academics and practitioners should simultaneously consider the impact of age, migration, and gender.

Originating from Black feminist scholars, intersectionality is understood as the critical insight that dimensions such as race, class, gender, nation, and age “interlock to create reciprocally constructing phenomena,” shaping complex social inequalities (Collins 2015, 2; Crenshaw 2017). Applying intersectionality to this case, I contribute to the ongoing literature acknowledging that adolescent migrant girls have distinctive reproductive realities that sit at the intersection of age, gender, and migration. While individual subjectivities are heterogeneous, the intersection of age, gender, and migration has been shown to contribute to gaps in SRH education, difficulties navigating health systems, as well as an increased risk of sex and gender-based violence in other contexts (Tirado et al. 2020; Korri et al. 2021; Ivanova et al. 2018; Bwambale et al. 2021; Jennings et al. 2019). Here, I explore what is being done to respond to the intersectional reproductive realities of Venezuelan adolescent migrant girls aged 15–19.^3^

## Using Reproductive Justice to Examine the Contraceptive Care of Adolescent Migrant Girls

In addition to the empirical data, this article offers a valuable addition to the existing literature by utilizing the RJ framework to assess the degree to which responses adequately address the unique and intersectional needs of adolescent migrant girls.

Conceptualized in 1994 by Black feminists from the Combahee River Collective, RJ adopts a more holistic understanding of SRH than a purely rights-based framework. Dissatisfied with the “right to choose” whether to have children espoused by white liberal feminist agendas, RJ was devised by Black feminists in recognition of the unique struggles of African American women. Combining legal rights and social justice movements, RJ is characterized by three main principles: “1) the right not to have a child; 2) the right to have a child; and 3) the right to parent children in safe and healthy environments” (Ross and Solinger 2017; Ross 2018). As a more capacious concept, RJ recognizes how axes of oppression intersect to stratify rights unevenly across populations (Morison 2022; Ross and Solinger 2017; Abji and Larios 2020). Subsequently, RJ centers historically marginalized groups in responses to redress power relations (Ross and Solinger 2017; Ross 2018).

Recently, RJ scholars have shown how actors’ responses (re)produce intersectional oppression to marginalize young people or migrants in a world that is primarily designed for adults and citizens to meet their needs (Hans and White 2019; Abji and Larios 2020; Wegelin et al. 2022). I use a RJ lens to expand this literature on the ICPD agenda’s commitment to contraceptive care of adolescent migrant girls in humanitarian crises. In this sense, failure to provide contraceptive care is not only a violation of the legal rights articulated in the ICPD Agenda. It is also a failure to redress the power imbalances that (re)produce reproductive injustices, which are embedded into crisis response.

Originating in the USA, the RJ movement has gained international attention (Chiweshe et al. 2017). Nevertheless, support is not universal. In Argentina, many “green wave” feminists who succeeded in using rights-based rhetoric to achieve advances, such as abortion reform, feel a new framework like RJ is unnecessary (Fernández Anderson 2020; Morgan 2015). However, it is important to acknowledge the heterogeneity of Latin America, where progress in reproductive rights has been far from linear (Friedman 2018).

Rights are a dynamic concept in Latin America, used to argue for and against the SRHR of women and girls (Fernández Anderson 2020; Arguedas-Ramirez and Wenner 2023). The advancement of women’s rights depends on the relationships between religious forces, social movements, state coalitions, and the democratization process (Htun 2003; Fernández Anderson 2020, 2022; Elgar 2011). Latin American SRHR and RJ advocates are united in their desire for social transformation, recognizing that SRHR is not evenly realized for marginalized populations (Arguedas-Ramirez and Wenner 2023; Nolan 2022). However, RJ encompasses more than access to abortion, contraception, and maternal care. RJ activists in the USA might focus on police brutality or a national living wage (Ross and Solinger 2017). Meanwhile, RJ advocates in Latin America address Global North–South relations, indigeneity, extractivism, and conflict—to which I add crisis response practices (Arguedas-Ramirez and Wenner 2023; Nolan 2022). RJ is, therefore, more adept than rights-based models at understanding how actors are designing and implementing responses to the intersectional reproductive realities of adolescent migrant girls, whereas a rights-based model may be better at examining legal reform or access.

In keeping with RJ literature, I view contraceptive care as one aspect of a continuum of SRH. Here, “contraception” refers to modern contraceptives as defined by the World Health Organization (WHO) (2018).^4^ The emphasis on contraceptive care—as opposed to contraceptive access—builds on RJ’s understanding of both the use and non-use of contraception as positive outcomes (Senderowicz 2020; Cadena et al. 2022). Thus, I reject the usage of “family planning” given its association with “choice” paradigms and neo-Malthusian population control, which can exclude adolescent girls and perpetuate inequalities (Rodriguez et al. 2014).

## Global Reproductive Health Inequalities

Black feminist scholars and demographers have critiqued how public health development and humanitarian responses interact to reveal a continuum of racialized, gendered, and colonial violence—or forms of reproductive injustice. I build on these claims to argue that seemingly “urgent,” technoscientific, apolitical responses naturalize the exclusion of marginalized populations such as adolescent migrant girls and their rights to access comprehensive contraceptive care.

Reproductive governance, developed by Morgan and Roberts (2012), explains how governments, institutions, and actors exert control over various aspects of reproduction. Reproductive governance can take many forms. For example, Bridges (2011) shows how, in obstetric care, racial prejudice leads providers to blame patients for receiving inadequate treatment. Meanwhile, Heller and Hannig (2017) show how surgical interventions to obstetric fistula are framed as a medical solution to a “cultural issue,” overlooking the structural injustices that cause them. Critiques of biomedical or technical practices such as obstetric violence highlight how discriminatory practices are steeped in racial and gendered discourses (Smith-Oka et al. 2022). Bridges (2011), Shim (2014), and Heller and Hannig (2017) have revealed that poor health outcomes are normalized as “cultural differences” between individuals rather than structural violence against “racialized others.”

In terms of contraceptive care, feminist science and technology studies draw attention to the process by which uneven or “selective” provision of technologies, such as long-acting reversible contraceptives (LARCs), exerts usually hidden forms of reproductive control over marginalized groups (Brandao and Cabral 2021; Brian et al. 2020; Gomez et al. 2014; Mann 2022; Morison 2023; Winters and McLaughlin 2019; Bhatia 2018).

Discourses of “urgency,” “cost-effectiveness,” and “rationality” are used to justify applying pressure for marginalized groups to use LARCs (Brian et al. 2020; Eeckhaut and Hara 2023; Morison 2023). LARCs are rationalized as a “good choice” for adolescent girls whose sexuality is often depicted as a risk to themselves, others, and the larger society (Morison 2023, 545; Brandao and Cabral 2021; Mann 2022). Risks are often based on normative expectations with particular concern for young, unmarried, poor, and minority patients’ reproductive behavior (Eeckhaut and Hara 2023, 4).

Poor women of color in the Global South have frequently experienced pressure to limit their family size in the name of overpopulation, environmentalism, economic development, and “modernization” unevenly compared to their white Northern counterparts (Hartman 1987; Hendrixson 2018; Nandagiri 2021; Brunson 2020; Murphy 2017; Bhatia et al. 2019; Kuumba 1999). Global health and development programs discursively reproduce normative “sexuality” through “rational projects” that appear neutral but are laden with moral assumptions (Corrêa et al. 2008; Pigg and Adams 2005). Pigg and Adams (2005) argue that interventions from the Global North in the Global South create “pathologized practices that violate the moral standard.” They thus demonstrate how international patterns of reproductive governance from policymakers and decision-makers in the Global North exert control over the bodies of women in the Global South.

Metrics, targets, and indicators operationalize biomedical technoscientific approaches that assume numbers are neutral and objective (Adams 2016; Pigg and Adams 2005). However, applying these measures to women and girls in the Global South often shapes healthcare practices (Adams 2016; Brunson and Suh 2020; Senderowicz 2019). Donor-driven priorities in global health have resulted in a narrow, top-down agenda focused on measurable indicators, as neoliberal actors push for metrics that demonstrate financial accountability and return on investment, prioritizing profits and health outcomes over broader needs (Storeng and Behague 2017, 164; Brunson and Suh 2020).

Metrics can obscure real reproductive challenges, as demonstrated in Senegal where abortion is illegal, and thus clinical record-keeping practices presented induced abortions as spontaneous abortions to avoid criminalization (Suh 2020; 2021). In obscuring the gaps in abortion care, health practitioners manipulate the reproductive realities of women on the ground (Suh 2020, 2021). This approach may compromise the quality of care, countering the ICPD agenda (Nandagiri 2021; Suh 2021; Bendix et al. 2019).

Family Planning 2020 (FP2020) is another example of a target-driven population policy that applies contraception as a “technical fix” to the problem of maternal mortality (Bendix et al. 2019; Brunson 2020; Hendrixson 2018). One target is to ensure 120 million more people from the poorest 69 countries are using modern contraception by 2020 (Bill and Melinda Gates Foundation and UK Aid 2012). In FP2030, this target applies to all countries but with a particular focus on low-income and lower-middle-income countries (FP2030 2022). The emphasis on targets for increased contraceptive coverage overlooks structural conditions that create reproductive injustices, such as obstetric violence and unsafe abortion.

Likewise, state actors, such as US President George W. Bush, utilized women’s and girls’ reproductive health as a foreign policy tool to ensure domestic support from conservative forces (Corrêa et al. 2008, 38). First established in 1984, the Mexico City Policy, or the Global Gag Rule, is a US foreign policy that prohibits spending projects that “promote abortion services.”^5^ Aside from reducing funding, the on–off evocation of the Global Gag Rule has prompted organizations to restrict activities to avoid accusations of non-compliance, ultimately increasing unintended pregnancies and births (Mavodza et al. 2019). In Colombia, the re-instating and re-traction of the Global Gag Rule three times in 30 years has had a significant impact on family programs, leaving tens of thousands without SRH care, particularly in rural areas (Daigle et al. 2022).

When donors favor certain activities over others—as seen in the Global Gag Rule—it creates inequalities between crises and groups within crises. In deciding who should receive that care, actors morally judge who is “deserving.” For example, “HIV exceptionalism” in Sierra Leone created “siloed” interventions that favored HIV + individuals (Benton 2015). Indeed, “womenandchildren” from the Global South are the ideal “passive victims” of Western aid (Enloe 1993; Spivak 2015; Carpenter 2005). This reproduces harmful racialized and gendered discourses that “refugee women” are one homogenous group “in need of saving” and “grateful” for any aid to be given (Crawley 2022; Mohanty 2019). Meanwhile, those who fail to conform are framed as “underserving of rights” (Morgan and Roberts 2012).

In humanitarianism, selectivity is justified through appeals to emergency or “urgent care” (Sulley and Richey 2023). However, the “global crisis approach” models have been criticized for being short-term, problem-centered, and operating in narrow silos (Foley and Hendrixson 2011). Applying a gendered focus, the concept of the “tyranny of the urgent” argues that actors set aside structural issues in favor of addressing immediate biomedical needs (Smith 2019; Davies and Bennett 2016; Watson and Mason 2015). The “urgent” is conceptualized relative to “later,” with “later” referring to the “patriarchal time zone” where women are asked to put their hopes for “the greater good” (Enloe 2004, 215). As such, the “tyranny of the urgent” demonstrates how the rights of marginalized groups fail to be upheld by responses that neglect social dimensions of gender and individual experiences (Smith 2019). Even if the aim is to “save lives,” overlooking structural aspects such as gender means accepting that some parts of the response will fail to reach women and girls (Eklund and Tellier 2012, 593). Consequently, actors’ responses are framed in “single axis” terms that discount gendered forms of oppression experienced at the intersections of age, migration, and gender (Bastia et al. 2022; Laurie and Petchesky 2008; Singer 2019). I envision these concepts as a spectrum of “urgent” to “later,” which is useful for understanding how intersectionality and contraceptive care have been hierarchized and deprioritized during crisis response.

## Methods

I combine a policy summary with qualitative interviews to explore how state and non-state actors have responded to the contraceptive care needs of Venezuelan adolescent migrant girls in Colombia. The policy analysis maps international and national-level obligations, while interviews provide evidence about their implementation.

I selected SRH-related policies by searching policy databases and correspondence with the Colombian Ministry of Health and Social Protection (MSPS).^6^ Key policies concerning the SRHR, adolescents, or Venezuelan migrants were added to a matrix (Appendix 1).

The main contribution of this article is the multi-perspective interviews conducted during fieldwork in Bogotá, Colombia, in March and September 2022. Thirty interviews were conducted, alongside three informal interviews. I used purposive sampling to recruit adolescent migrant girls as well as medical professionals, national and local public health and social protection professionals, civil society organizations, humanitarian organizations, and social welfare experts working to design and implement SRH policies and programs for Venezuelan migrants (Appendix 2).

Adolescent migrant girls contributed experiential knowledge of reproductive realities. Purposive sampling was used to locate and interview adolescent migrant girls because this group is relatively understudied. This resulted in some limitations regarding the diversity of the sample. To get a fuller picture, future research should focus on more rural populations, more racial and ethnic minorities, non-cis-gendered, non-heterosexual adolescent migrants.

Meanwhile, exploring the attitudes and practices of key informants achieved the article’s main aim: to understand how state and non-state actors consider adolescent migrant girls in the design and implementation of their responses. Most key informants were based in Bogotá. Nevertheless, many had worked in other cities such as Barranquilla, Bucaramanga, Medellín, Calí, Cartegeña, Cúcuta, Riohacha, and Soacha. This was beneficial for comparing between departments. However, rural populations are less represented.

Interviews lasted an hour in English or Spanish. An interpreter was present for Spanish interviews. Data analysis consisted of transcription, pseudonymization, two rounds of coding, and a mix of inductive and deductive thematic analysis. A total of 36 codes were identified, and key quotes were selected as examples for discussion.

The research was approved by the University of Southampton (ERGO 70067 and ERGO7081) and Profamilia (CEIP-11–2022). Adolescent migrant girls aged 18 or above were interviewed through a gatekeeper organization. They spoke about their experiences retrospectively, having arrived in Colombia between 2018 and 2022, while aged 15–19. Additional protocols included separate consent forms, participant information sheets, and interview guides.

## The Policy Landscape of Venezuelan Adolescent Migrants’ Contraceptive Care in Colombia

This passage briefly reviews the international and national policy frameworks concerning the contraceptive care of adolescent migrant girls in Colombia. The key policies reviewed include the ICPD Program of Action, the ICPD Beyond 2014 and ICPD + 25, the Minimum Initial Service Package (MISP), and Colombia’s National Policy on Sexual and Reproductive Health Rights Plan (from here on Colombia’s SRHR Plan).

State actors are principally responsible for fulfilling SRHR as outlined in the ICPD Program of Action. Later versions of the 1994 ICPD Program of Action, the ICPD Beyond 2014, and later the ICPD + 25, included calls to provide modern contraceptives, adolescent-friendly services, and “to ensure basic humanitarian needs and rights” in humanitarian settings (UNFPA 2016, 2019a). Although the complexities of age, gender, and displacement in humanitarian contexts are acknowledged, the ICPD agenda's creation of separate commitments for adolescent girls and migrants results in reduced entitlements for migrants during crises, precisely when they need them the most.

Established by the Inter-agency Working Group on Reproductive Health in Crises, MISP is a set of guidelines articulating the scope of “minimum, lifesaving SRH needs” to reproductive healthcare in humanitarian crises. At the onset of a crisis, MISP recommends providing intrauterine devices (IUDs) “to prevent unwanted pregnancies” and condoms for HIV prevention (IAWG 2018). After 6 months, crisis-affected persons should have access to comprehensive contraceptive care. Until then, other contraceptive methods and adolescent-friendly health care are “additional priority actions” outside the scope of the initial response (*Ibid*.). MISP thus creates a hierarchy in which some forms of SRH are prioritized in a designated period of “crisis,” during which some aspects (e.g., adolescent girls and contraception) are compartmentalized for “later.”

Turning to Colombia specifically, the response to Venezuelan migrations has been understood in terms of state and non-state actors. Non-state actors predominantly operate through the Interagency Group of Mixed Migration Flows (GIFMM). GIFMM was established in 2016 by the United Nations High Commissioner for Refugees (UNHCR) and the International Organization of Migration (IOM) as the national leaders of the regional response to the Venezuelan migrant crisis (R4V) (R4V 2021b). With 75 members, including the Colombian MSPS, GIFMM coordinates working groups or Clusters. This Cluster Approach aims to distribute responsibility for providing health, shelter, and other services among various agencies (UNHCR 2024). Currently, the IOM and WHO lead the Health Cluster, providing financial aid and technical assistance to improve available services and bridge gaps in the healthcare system, and which houses the sub-cluster on SRH co-led by MSPS and the UNFPA.^7^

Implemented by the sub-cluster on SRH, the Colombian version of MISP remains limited to the priority areas addressed in the international version (UNFPA 2019b). Unsurprisingly, “additional priority areas,” such as other contraceptive methods and adolescent-friendly services, are not mentioned. Consequently, they obscure two key aspects of SRH, which Venezuelan women and girls have articulated they want (Flórez García et al. 2020; Profamilia 2019).

The Colombian Government articulates guidelines for implementing the ICPD Program of Action in Colombia’s SRHR Plan (2014–2021) (Profamilia et al*.* 2014). The finer details reveal limited obligations toward migrants. Equally, the adolescent and youth-friendly health services model (*servicios amigables*) refers only to Colombian citizens, leaving migrants excluded. It is best, therefore, to turn to the recommendations on integrating Venezuelan migrants into the national health system to understand adolescent migrant girls’ entitlements to contraceptive care.

Since 2017, policy developments expanded Venezuelan migrants’ access to healthcare (Appendix 1). Prominent changes have included routes for the affiliation of Venezuelans to the health system and access to emergency care regardless of status (External Circular No.25 of 2017) (Ministerio de Salud 2017). Meanwhile, those unaffiliated were encouraged to regularize their status through the Temporary Statute of Protection (EPTV) scheme, granting temporary status to Venezuelans from 2021 to 2031 (Migración Colombia 2021). Yet, critics have challenged the state discourse of Colombian generosity towards Venezuelan migrants (Palma-Gutiérrez 2021). Legal scholars have questioned whether the protection afforded to young people from Venezuela in Colombia satisfies the responsibilities of the state as a signatory to the United Nations Convention on the Rights of the Child, International Human Rights Law, and the Inter-American Human Rights Law (Pelacani 2022; Angeleri 2021).

A more gender- and age-sensitive approach to Venezuelan migrant health care may be on the horizon. In August 2022, External Circular 35 of 2022 recommended that departments provide comprehensive contraceptive methods regardless of migration status (Ministerio de Salud 2022). Objective 3.4 of External Circular 35 takes a “gender and differential approach” to protect the SRHR of Venezuelan migrant girls. This includes promoting free access to modern contraception methods, especially among young people and those in an (ir)regular situation. The impact of External Circular 35 is yet to be determined. However, given the findings below, we have reason to be cautious about the extent to which this will be successful.

While policies in Colombia are currently supposed to apply a “gender focus” and “differential approach,” binding policies on Venezuelan migrants have shown little regard for variation within the migrant population. The most progressive legislation is non-binding, relying on whether and how local actors choose to implement these recommendations. For the most part, Colombia produces two different responses: one for Venezuelan migrants and one for Colombian adolescents. These “single-axis” approaches that focus on migrants, women, adolescents, or migrants as age-less or gender-less are precisely what RJ as an intersectional framework seeks to overcome (Abji and Larios 2020; Ross 2018). Within these silos, adolescent migrant girls’ obligations are reduced to “basic” or “lifesaving” needs on account of their non-citizenship. To prevent the state from shifting its responsibilities onto adolescent migrant girls for their lack of access to SRHR, policies should acknowledge the complex realities these girls face and develop appropriate responses accordingly. Failing to recognize adolescent migrant girls as rightsholders who currently experience intersectional disadvantage further marginalizes them, creating reproductive injustices.

## Interviewees’ Perspectives on Responses to Contraceptive Care for Venezuelan Adolescent Migrant Girls

Understanding that the design of policies and their implementation are two separate processes, this section draws from the multi-perspective interviews to ask how actors implement responses that align with the intersectional reproductive realities of Venezuelan adolescent migrant girls.

### State-Led Public Health Responses

When asked how they adapted their practices to consider the contraceptive care needs of adolescent migrant girls, state actors first pointed to the regularization of Venezuelan migrants into the health system (via the EPTV). Additionally, several public health and medical professionals underscored the importance of the provision of emergency care (External Circular No.25 of 2017). The EPTV provided a basis by which Venezuelan migrants would be entitled to “the same care as Colombians” (Victor, a public health professional).This legislation aimed to address one of the key barriers that prevented service providers from delivering contraceptive care—a lack of access to the healthcare system due to migrants’ status. Without regularization, Venezuelan adolescent migrant girls were refused access to contraceptive methods.

*“[I]f they’re a migrant with regular migration status, then we can set them up with a roadmap for care. But if they have an irregular status, we can’t link them to the health system.” Liliana, a public professional working in social protection.*

Likewise, Venezuelan adolescent migrant girls saw affiliation to health insurance as the “biggest barrier” to accessing contraceptive care. As Alejandra articulates, without access to health insurance (EPS), the cost of SRH care, including contraceptive care, is too expensive.

*“If you have an EPS, then you can ask for an appointment; you could go see the gynecologist […] if you don’t have that, then you have to get private care, and it’s too expensive.” Alejandra Venezuelan migrant living in Bogotá.*

However, the complexity of the process meant the EPTV scheme was not operating as designed. Those foundations and NGO workers who consulted with migrants in their everyday roles (providing healthcare services or orientation to healthcare services) noted bureaucratic hurdles to regularization. For example, the application required documentation such as a passport “that many Venezuelan migrants don’t have” (Carmen, a public health professional). To address this issue, many non-state actors delivering humanitarian aid had a legal expert to help Venezuelan migrants like Alejandra with the EPTV process.

The second way actors stated they met the contraceptive care needs of adolescent migrant girls was through access to “emergency” or “urgent care” (External Circular No.25 of 2017). While in theory, this policy was to be implemented universally, health providers, local foundations, international NGOs, and adolescent migrant girls described it as “not effectively implemented for migrant women” (Laura, a professional from an international non-governmental organization (INGO)).

Drawing on her experience, Alejandra recounted seeking legal assistance from another organization. She filed a constitutional writ (a *tutela*) to secure access to maternal care. When she received care, she specified that it was for her son's health and not her SRH. As such, there was no offer of postpartum contraceptive care or gynecological check-ups.

*“[I]t’s not that I could get a gynecological check-up or talk to anybody about my health as a woman, it’s that I was able to get in the door because I was pregnant.” Alejandra, a Venezuelan migrant living in Bogotá.*

In contrast, Daniela (another Venezuelan migrant living in Bogotá) had been denied publicly funded prenatal care altogether. She could afford one ultrasound from a private health provider but no further care. Key informants’ experiences confirmed that Daniela’s case was common. Medical providers, multilateral service providers, and civil society professionals stated that irregularity created barriers such as security guards refusing entry, administrative staff’s inability to process payments, and variation in perceived “urgency.” Daniela and Alejandra’s experiences serve to demonstrate how unevenly legally binding SRH policies are applied to Venezuelan adolescent migrant girls.

Although contraceptive care is not considered “urgent” care, these experiences served to contextualize the realities in which Venezuelan adolescent migrant girls are seeking SRH. Further, given that existing legally binding legislation is implemented unevenly, what effect will new non-binding recommendations have on providing contraceptive care to irregular migrants? The interviews demonstrate that access to any form of SRH care is primarily decided through migration status. Making care conditional on affiliation to the health insurance system while overlooking the barriers to affiliation demonstrates how Venezuelan adolescent migrant girls have not been included in state responses to their contraceptive care.

### Humanitarian Responses

As the health system did not provide contraceptive care to irregular migrants, many migrants turned to not-for-profit organizations, multilateral organizations, NGOs, and civil society providers. These organizations were supposed to “help fill gaps” in healthcare for the irregular migrant population while they became affiliated (Maya, a multilateral program designer, and Victor, a public health professional).

Humanitarian non-state responses were characterized by their short-term approach. The number of responses delivered via short-term projects led many participants, like Laura, to characterize the response as “reactive.”

*“There is a humanitarian situation. So humanitarian aid has taken place, and that is important, but what we see is that the responses are being implemented from a crisis perspective, not with permanent solutions.” Laura, a legal professional from an INGO.*

While attention had been focused on immediate relief efforts, there was a lack of comprehensive, long-term planning to address the underlying problems. Providers such as Hector (a professional from a not-for-profit medical service provider) explained that many Venezuelan adolescent migrant girls were “not asking for emergency care” but long-term services such as contraceptives. Short-term approaches limited the availability of follow-up care, as in the case of Clara.

*“I first went to that organization for that reason asking for resources and they helped me receive an abortion […] then after that process was over at the last session, they offered me different contraceptives. They gave me a few minutes to think about it and I decided on that one given all the options because it seems like the most effective to me.” Clara, a Venezuelan adolescent migrant girl living in Bogotá.*

Clara had an IUD fitted as part of post-abortion care from an international humanitarian organization. Regrettably, she could not recollect the organization’s name and had lost contact with them, precluding the possibility of scheduled removal check-ups to monitor the condition of the IUD. Equally, key informants from (I)NGOs and not-for-profits raised concerns about the ethics of one-off interventions that did not provide follow-up care. Namely, there was no post-insertion follow-up or sustained availability of healthcare services in case of complications or a desire for removal, as recommended by established guidelines (WHO 2018). These findings elucidate how the responses continued to be driven by traditional humanitarian risk-reduction, disease-based frameworks rather than comprehensive responses, which included care responsive to the intersectional needs of adolescent migrant girls. As I will later discuss, without follow-up care to monitor or remove LARC responses, one-off interventions can lead to longer-term reproductive injustice.

Other times, state and non-state actors coordinated to establish humanitarian responses to address “priority issues” (Julia, a public health professional). They established “packages” to divide care into categories. Women’s SRH was largely considered in terms of maternal care and sexual and gender-based violence, while healthcare for children focused on vaccines, nutrition, and education. Consequently, key informants organized healthcare needs by population, leaving adolescents’ contraceptive care needs to fall between the silos of women’s and children’s health. One reason Victor (a public health professional) contributed this to the fewer numbers of adolescent migrants compared to the wider migrant population.

*“But we have not put so much emphasis on adolescents because we know that they are a priority population, vulnerable, but they are not the majority of the population.” Victor, a public health professional.*

This reflected the ongoing pattern of de-prioritization of the needs of Venezuelan adolescent migrant girls.

The MSPS contracted local healthcare providers or civil society actors focusing on SRHR to implement centrally defined packages. This approach ensured that care was delivered by an actor with a pre-existing relationship with the Venezuelan migrant community, increasing the likelihood of success in the implementation process. The local departmental health authorities were responsible for overseeing the implementation of these packages according to the needs of that geographic territory. The central government had “requested” that local authorities “differentiate” their territory-specific development plans (*Collective Intervention Plans*) to “include Venezuelan migrants” (Carlos, a public health professional). Commenting on the feasibility of this, Carlos continued, “I’m not going to say that this is systematically carried out. It depends on the local government.” Based on the variation in quality and quantity of responses between departments, it became clear that centrally devised recommendations were not consistently implemented.

Regional variations were due to some gaps being “easier” to address than others.

*“Unfortunately, not all [international organizations] provide the necessary number of check-ups or all the tests. […] There’s a lot of oversupply [sic] because all three [out of 14] offer pretty much the same services.” Carmen, a public health professional.*

In this case, Carmen refers to the oversupply of maternity care, which exemplifies the prioritization of maternal care over other forms of care. This oversupply of some services, while gaps remain among others, aligns with the hierarchical structure observed in state and non-state responses, where emergency or life-saving care took precedence.

Differences in the type of care provided, who it is for, and the duration of care were accompanied by inequalities in accessibility determined by location. Each territory had a humanitarian response tailored to its specific context, depending on the focus of the local departmental health authorities and the assistance they received from international donors. This ensured the response was adapted to the region’s (lack of) infrastructure and the high numbers of migrants with chronic health needs. However, it also generated disparities between departments when responses were only delivered in areas classified as “urgent,” such as border towns (Catalina, a lawyer from an NGO).

*“I feel that the discussion about Venezuelan migrants, starts in Cúcuta, and stays there.” Laura, a lawyer from an INGO.*

While the prioritization of services for the migrant population is essential, the unequal access to contraceptive care for some groups, depending on time and place, highlights the substantial gaps in the provision of contraceptive care to Venezuelan adolescent migrant girls. Respondents like Carlos (a public health professional) were quick to generalize their answers to describe services that were available for “not just adolescents but the Venezuelan population in general.” What, then, prevented actors from differentiating services to include Venezuelan adolescent migrant girls?

### Lack of Differentiated Responses to Adolescent Migrant Girls’ Contraceptive Care

First, actors relied heavily on donors to fund programs. Key informants pointed out that donors—whether international multilateral organizations, foreign national governments, or philanthropic individuals—often imposed predetermined ideas on which activities should be supported, constraining the implementation of contraceptive care responses. Program designers frequently cited the prohibition on abortion-related activities (which are intermittently expanded and rescinded under the Global Gag Rule) as forcing them to choose between accessing funding from the USA and offering comprehensive services (Manuel, a professional from a multilateral organization; Isabel, a professional from an NGO; and Fernando, a state integration professional). Equally concerning, other donors with conservative ideologies sought to provide LARC without offering sexual education, demonstrating that the donors’ focus on indicators aimed at reducing adolescent pregnancy rates overlooked the SRHR of adolescent migrant girls.

*“[…W]e’ve even had some donors that want to give contraceptives to all the girls and didn’t really care about the education behind it. They just like to put some contraceptives for all so that they don’t get pregnant […] When they realize that we talk about abortion and that we are a feminist organization- they withdraw.” Isabel, a professional from a not-for-profit organization providing education.*

While some actors managed to negotiate broader service offerings, others narrowed theirs to exclude topics donors considered “controversial.” Trade-offs were made between the different aspects of SRH, with those central to the agenda gaining more attention than others. Building on her point above, Isabel recounted how she secured funding for a water, sanitation, and hygiene (WASH) program, which included education on menstrual health but not contraceptive care. Aside from the Global Gag Rule, the diversion of resources due to events such as COVID-19 and the Russian-Ukrainian war exacerbated the lack of funding for Venezuelan migrants’ SRH.

As Hector (a health program designer for a not-for-profit organization) remarked, the project's scope “depends on the donor’s logic of monitoring and evaluation” that insisted program activities align with strict criteria. For instance, donors refused to provide abortion services or medicines produced by pharmaceutical companies outside the USA. Others restricted their contracts to specific cities despite a need for services between and around these cities. The need to follow pre-established contracts made it difficult to respond swiftly to the rapidly changing needs of the population, reinforcing a top-down approach.

“Technical” and “emergency” framings of crisis responses are underpinned by moral judgments about which activities are acceptable and worthy of funding. These frameworks resemble the “rational projects” conceptualized in the literature on inequalities in global reproductive health (Brunson and Suh 2020; Corrêa et al. 2008). Here, monitoring and evaluation frameworks prioritize the counting of indicators and fulfillment of contracts, making invisible service needs that are less desirable to donors, such as adolescent SRH and abortion, regardless of their importance to migrant communities.

Second, there was a notable absence of adolescent-friendly health services. Overall, key informants like Isabel (a professional from a not-for-profit organization) saw the adolescent-friendly health services as a strong policy but “hard to implement” into the health system—even for members of the host population. This finding is supported by Huaynoca et al. (2015), who discovered that a lack of trained health personnel, variation between departments, and negative perceptions among community members hindered coverage. For many of the Venezuelan migrant population who experience a lack of access to sexuality education, have higher rates of adolescent pregnancy, and often have few resources to spend on private or out-of-pocket contraceptive care, the lack of access to adolescent-friendly services creates clear violations to SRHR in the ICPD and the principles of RJ.

*“[I]f we’re working here from our clinic, we have youth-friendly spaces, for example. That’s not as easy when we’re offering extramural or mobile care.” Luis, a professional from a not-for-profit organization providing healthcare.*

Key informants largely focused on the difficulties in providing these services to adolescents in the host population, revealing a lack of adaptation for adolescent-friendly health services for the migrant population. Despite efforts by non-state actors to offer adolescent-friendly services in humanitarian responses, challenges persisted. For example, the adolescent-friendly health services model articulates the need for adolescent-friendly spaces and private consultations. Luis’ quote above shows how some service providers operated through temporary or mobile units where such operations were not feasible. In short, the adolescent-friendly health services framework was differentiated for adolescents but not for migrants; meanwhile, the migrant responses address the needs of Venezuelans but not adolescents. The absence of adolescent-friendly, migrant-inclusive health services further reinforces the single-axis approach and highlights the fragmentation of services. When services are fragmented, adolescent-friendly health services are often among the first aspects to be neglected.

Third, the lack of information emerged as a prominent barrier, with participants highlighting that “migrants may arrive and want to have access to contraceptives, but they don’t know where or how to ask for them” (Maria, a lawyer from an NGO). Adolescent migrant girls, like most migrants, lacked the information on how to access healthcare in Colombia; however, this barrier was compounded by a lack of knowledge and experience in seeking SRH.

*“[I]t’s really difficult especially for adolescents when they change a setting or change their home because especially because we don’t know a lot about that topic.” Clara, a Venezuelan migrant living in Bogotá.*

The high number of fractured and overlapping services made navigation difficult. Clara relied on civil society actors for guidance on available services because there were “so many programs and you hear so much information and sometimes it’s overwhelming.” It was common for Venezuelan migrants to approach asking “for any humanitarian help they could offer.”^8^ The call for clearer information demonstrates a need for adolescent-friendly communication strategies.

Finally, where information was provided, it failed to acknowledge that the perspectives of Venezuelan adolescent migrant girls may differ from the host population.

*“[Venezuelan migrants] use different words for condoms than we do in Colombia, and I think that on the other hand, there’s a cultural influence they see family planning […] as a family issue. It’s not something that you talk about in public.” Paola, a health professional for a charitable foundation.*

For many, family planning meant “planning to have a child” (Leila, a professional working for a not-for-profit). Euphemistic approaches to “family planning” (or *la planificación*) serve little purpose other than to situate contraceptive care within the notions of heteronormative families. Likewise, “family planning” programs fail to promote contraceptive care for non-contraceptive benefits. Concealing contraceptive care in the language of “family planning” overlooks the fact that many Venezuelan adolescent migrant girls did not want to have a child soon or were not in a union. This emphasis on contraceptive care solely for reproduction decouples the provision of medical resources from the broader goals of providing comprehensive education and information.

Comprehensive education and information, regardless of the decision to use or not use contraceptive methods, are essential for the development of adolescents’ health outcomes and ability to navigate relationships. The absence of opportunities for education and information is a barrier to informed contraceptive decision-making that is shaped by age. Therefore, it is crucial to recognize that current responses do not address the specific challenges faced by Venezuelan adolescent migrant girls. Failure to do so risks framing unequal access as an immutable characteristic of adolescent migrant girls, overlooking the unique ways in which structures of gender, age, and migration have shaped responses that discourage access to contraceptive care.

## Discussion

This article began by asking how actors responded to adolescent migrant girls’ contraceptive care, using the case study of Venezuelan migrants in Colombia. RJ advocates for a comprehensive system of care across the continuum of SRH and the environments in which they are sought. Above all, RJ focuses on providing encouraging environments for the most marginalized groups. In this case study, I found a lack of intersectional responses that simultaneously account for gender, age, and migration. Instead, Venezuelan adolescent migrant girls’ contraceptive care is largely shaped by their identities as migrants. This discussion synthesizes the policy review and multi-perspective interviews to demonstrate how state and non-state responses serve to fragment the continuum of SRH into silos. Throughout this discussion, I consider the findings through the lens of RJ, highlighting how adolescent migrant girls are discouraged from accessing contraceptive care by the “tyranny of the urgent,” which prevents longer-term intersectional responses from being implemented.

The policy review found that at both national and international levels, the post-ICPD agenda has addressed “family planning” or contraceptive care as an issue separate from adolescent-friendly services and SRH in humanitarian crises. The latter only requires access to “basic” services, which means migrants are entitled to a lower minimal standard than citizens. There is no emphasis on the intersectional components that may involve adopting a migrant-inclusive, adolescent-friendly health service. Already, the political and legal structures of reproductive governance that shape the agenda normalize the deprioritization of adolescent migrant girls’ contraceptive care. Instead of intersectional responses, they focus on what feminist international development literature terms “technical solutions” to reproductive health “problems” (Bendix et al. 2019; Brunson 2020; Hendrixson 2018). Thus, while appearing neutral, they normalize the fragmentation and use of LARC over adolescent-friendly health services. Specifically, MISP’s emphasis on LARC alongside and deprioritization of adolescent-friendly services means that there is no legal mechanism by which to recognize “emergency-only” care as a type of reproductive injustice. The categorization of migrants as a separate group—who experience lower SRHR outcomes—shows that this case is another example of RJ as a matter of migrant justice (Abji and Larios 2020).

The findings from the interviews support these policy findings. While routes to affiliate with the health system for irregular migrants demonstrate a step toward improving contraceptive access, difficulties in acquiring documents and the overly bureaucratic nature of the process disproportionately disadvantaged poorer Venezuelan migrants, who often required legal assistance to complete the process. This is consistent with existing literature on Venezuelan migrants’ right to health in Colombia (Pelacani 2022; Angeleri 2021). But what do these challenges for regularization mean for contraceptive care?

Contrary to the aims of the ICPD Program of Action, which seeks to address vulnerabilities hindering the realization of SRHR, the lack of effective and equitable implementation instruments poses additional challenges to achieving contraceptive care for adolescent migrant girls. The adolescent migrant girls interviewed stated they wanted more knowledge of SRHR and clearer information on seeking SRH care. Some arrived pregnant, seeking help with delivery, or seeking an abortion. This is consistent with the findings of Cintra, Owen, and Riggirozzi (2023), who demonstrated how the state response overlooks gendered aspects of migration, such as the fact that SRH care is a driver for migration. Building on the findings of Cintra, Owen, and Riggirozzi (2023), my findings show the immediate need for SRH is shaped by age, as adolescents often lack comprehensive sexuality education in Venezuela and have less experience in navigating the health or legal system (Ramírez-Martínez et al. 2023; Save the Children 2020). This experience differs from that of Venezuelan adult women, many of whom have had previous knowledge about contraception but who do not know how to access it (Flórez García et al. 2020). The failure to differentiate responses is to accept that the policy will be unevenly implemented and that the worst off will be further disadvantaged (Eklund and Tellier 2012, 593).

In some instances, contraceptive care constitutes emergency care, and in these cases, the law dictates that services must be provided regardless of status (External Circular No.25 of 2017) (Ministerio de Salud 2017). However, participants noted that access to emergency care was highly varied. If provided, care was specific to the emergency and did not include postpartum contraceptive care. There was frequent confusion because migrants often assumed their lack of care was a result of their lack of status, not of improper implementation. Once more, those without the knowledge or means to process a *tutela* and challenge their lack of SRH care, such as Daniela (who earlier recalled how she was denied maternal care), are discouraged from seeking contraceptive care.

Given the unpredictable implementation of External Circular No. 25 of 2017, there is little reason to think that recommendations such as Circular 35 of 2022 (which recommends providing contraceptive care to irregular Venezuelan adolescent migrant girls) will be implemented differently. Currently, no data has been published, although future research will hopefully give greater insight into the long-term impact of this policy. However, it further shows that despite the importance of legal policies, their effectiveness in practice is often shaped by their implementation.

Across Colombia, there are high numbers of adolescent migrant girls seeking contraceptive care who have irregular status and are not in “urgent” situations. These girls were mostly directed toward humanitarian service providers. This leads us to another key finding: coordinated responses between state and non-state actors are siloed and fragmented, driven by donors’ preferences. Within this hierarchy, adolescents and comprehensive contraceptive care are deprioritized in favor of narrow target-driven approaches that promote LARCs in the absence of a variety of methods.

State and non-state actors worked together on the response to Venezuelan migration, developing packages for priority areas for vulnerable groups. While this can be understood as a mechanism to promote international responsibility sharing, as is mentioned in the ICPD, the siloed approach often ended up fragmenting the continuum of SRH.^9^ The migrant population was categorized into broad age groups. So, healthcare needs were only established according to those predefined groups, failing to account for differences *within* groups. The categorization of “pregnant women and children” reflects a reductionist view of women in the Global South, as explained by Enloe’s (2004) concept of “womenandchildren.” Aggregating women and children into one homogenous group overlooks the intersectional experiences of adolescent migrant girls discussed throughout this article. As a result, adolescent migrant girls who straddle the space between not-children-but-not-yet-adults have their needs and preferences sent to the time zone of “later.” Without understanding how age impacts SRH inequalities, responses will continue to homogenize the reproductive realities of Venezuelan adolescent migrant girls with Venezuelan migrant women.

Program cycles, focusing on improving predetermined “priority” packages or “emergency care,” continued to legitimize short-term responses that overlooked both intersectionality and contraceptive care. Many participants felt the responses were stuck in short-term “crisis” interventions. Aligning with the documents reviewed in the policy summary, by now, the Colombian government and partner organizations should have provided contraceptive care for irregular migrant women and girls from Venezuela. Under MISP, 6 months after the onset of a crisis, access to comprehensive care should be implemented (IAWG 2018). Therefore, the continued emphasis on short-term emergency responses reflects the concept of the “tyranny of the urgent,” which prioritizes immediate biomedical needs and technical solutions over structural issues (Smith 2019; Davies and Bennett 2016; Watson and Mason 2015). The “tyranny of the urgent” demonstrates how the rights of marginalized groups and intersectional experiences are neglected by responses that make sources of inequality invisible (Smith 2019). This siloed approach disrupts the continuum of SRH by creating a hierarchy where some types of care are more valuable (emergency care) and can justify the absence of others (non-emergency contraceptive care). In this sense, the evocation of “crisis” and “emergency” is a means of maintaining the status quo and legitimizing the lack of intersectionality. In essence, by disregarding the complexities of intersectionality, these responses frame experiences of age, gender, and migration as less relevant to the current crisis, further marginalizing and excluding adolescent migrant girls.

Equally, certain types of SRH care were prioritized, meaning that groups seeking types of care had preferential access. As in Benton’s (2015) example of Sierra Leone, “HIV exceptionalism,” where HIV + patients were treated preferentially, certain issues received more attention. In Colombia, emergency SRH care was only offered to Venezuelans who were pregnant women, HIV + patients, or victims of violence. Contraceptive care was not in itself a standalone service. In Sierra Leone, HIV + status opened the door to a variety of healthcare services; in this case, care was limited to the specific condition on a one-time basis. This leads to a situation where some care is provided based on its “exceptional status” without consideration of the long-term impact of withholding non-emergency care. The risks of withholding contraceptive care include the risk of sexually transmitted infections and diseases, including HIV/AIDs, unintended pregnancy, and compromised bodily autonomy (Darroch et al. 2016).

The adolescent migrant girls interviewed in this study wanted access to information about contraceptive care—especially on how to navigate services. Likewise, informants recognized that terms like “family planning” (or *la planificación*) did not translate to girls not currently planning to have a family, who thus refused services that might have been relevant to them, like comprehensive sexuality education or access to contraceptives. Some individuals may be hesitant to openly discuss contraceptive care, a reluctance further compounded by providers’ use of euphemistic language. However, it is crucial to avoid using “cultural differences” as a pretext for holding Venezuelan adolescent migrant girls responsible for the lack of care they receive in the way that “racialized differences” are used to blame marginalized populations for poor health outcomes (Shim 2014; Heller and Hannig 2017; Bridges 2011). Instead, we need to acknowledge that such discourses constrain access to contraceptive care for adolescent migrant girls because they are couched in heteronormative euphemistic terms which assume a level of pre-existing knowledge from the service providers. This choice of language normalizes the lack of adolescent-friendly health services as a characteristic of difference rather than inefficient program implementation. Researchers and actors must develop innovative ways of providing adolescent-friendly health services in all circumstances, including effective and inclusive communication strategies, while ensuring consultations are conducted in privacy and with confidentiality.

Donors’ preferences were perpetuated by key performance indicators, which sustain a cycle of responding to needs based on the measurements deemed valuable by donors. This exacerbated hierarchies of SRHR where perceptions of need were evidenced by indicators, yet the data collection for indicators was often limited to “urgent” components of SRH. Hence, aspects such as adolescent-friendly services, contraceptive utilization rates, and unmet contraceptive needs were not part of needs assessments or subsequent programs. Response agendas evaluated needs according to the most “urgent” or visible aspects of SRHR, as defined by predetermined indicators. This short-sighted, fragmented, and indicator-driven approach led to an overly narrow focus on the most visible issues without addressing underlying causes. Consequently, program designers had to adapt their programs to match donors’ indicator preferences and neglect the rapidly changing needs of migrant communities. This manifestation of the “tyranny of the urgent” reinforces the idea that programs that focus on short-term, technological fixes are more appealing to donors.

Actors like Isabel, a professional from a not-for-profit organization, aimed to provide comprehensive education but faced challenges in convincing donors that initiatives without quantifiable technical solutions—such as knowledge production—were as valuable as outcomes like the “number of implants administered.” This is concerning considering debates in RJ about LARC hegemony or LARC-first approaches to consultation. For example, groups typically cast as “non-compliant” to normative understandings of “sexuality,” like adolescent migrant girls, are provided LARC to meet targets in public health and social reform agendas rather than enhancing autonomy (Brian et al. 2020; Morison 2023). Thus, the application of LARC without comprehensive education and information should be recognized as a form of reproductive control (Brandao and Cabral 2021; Brian et al. 2020; Gomez et al. 2014; Mann 2022; Morison 2023; Winters and McLaughlin 2019; Bhatia 2018). Furthermore, it contradicts the ICPD Program of Action, which aims to eradicate population control by transferring “the right to decide” to the individual and creating obligations for states to provide information and education, meanwhile discouraging coercive or discriminatory policies (UNFPA 2014). However, my findings demonstrate that the ability of adolescent migrant girls to make contraceptive decisions is controlled by international global health actors reproducing SRH inequalities in an already marginalized population. Therefore, my findings resonate with previous discussions of population control exerted via neoliberal business-like practices through vertically narrow, top-down responses implemented in the Global South by actors based in the Global North (Brunson and Suh 2020; Bendix et al. 2019; Bhatia et al. 2019; Hendrixson 2018; Murphy 2017).

Colombian actors faced further difficulties in acquiring international funding for topics like adolescent contraception and abortion—given the Global Gag Rule and the religious or conservative attitudes of major donors. Just as Enloe (2004) and Benton (2015) seek to explain why “womenandchildren” and HIV are high on donors’ agendas, Htun (2003) explains why some decision-makers avoid SRH topics perceived as controversial or “taboo.” Non-divisive elements often focus on components of SRH framed as “essential,” such as maternal and child health, rather than policies that aim to understand the reproductive realities and create long-term transformative change. This reflects the findings presented here: that certain programs are more appealing to donors. Consequently, humanitarian responses framed as technical assistance to “fill gaps” in reality employed moral agendas.

Moral agendas convey ideas about what sexual behavior is permissible. Most notably, religious fundamentalist groups rejected parts of the ICPD agenda that are seen as incompatible with their teachings—opposing adolescent SRHR because it “promotes” premarital sexual activity and denouncing abortion as “murder” (Pigg and Adams 2005; Corrêa et al. 2008). In the case of Venezuelan adolescent migrants in Colombia, the preferences of international donors to fund some of these topics over others have created a hierarchy where some interventions (e.g., WASH, maternal care) were preferred over others (contraceptive education, short-term contraceptive methods, adolescent-friendly health services, abortion services).

Framing some populations as (un)worthy of certain types of care perpetuates an atmosphere of fragmented accountability because it allows actors to be selective in their response. The “tyranny of the urgent” is used to hierarchize types of care to certain populations in which contraceptive care is de-prioritized. By distancing themselves from being accountable to non-emergency needs, humanitarian and state actors can legitimize their non-inclusion of contraceptive care and lack of intersectional responses for Venezuelan adolescent migrant girls. These responses reveal how the “tyranny of the urgent” operates as a form of reproductive governance by discouraging longer-term comprehensive care that would address intersectional reproductive realities.

This discussion ends by advocating for a RJ approach that considers contraceptive care as one aspect of the continuum of SRH. This includes acknowledging the reproductive realities of those with intersectional needs and taking actions to ensure responses are welcoming to their distinct needs. Currently, the fragmentation of SRH services creates hierarchies within the continuum of SRH so that contraceptive care of Venezuelan adolescent migrant girls is not encouraged, prioritized, or consistently addressed. To enhance the response, it is imperative to adopt intersectional comprehensive responses that illustrate how multiple social, cultural, and political factors shape who is encouraged to access contraceptive care. While short-term emergency care may be suitable at the onset of a crisis, situations of protracted crisis demand a holistic view that recognizes contraceptive care as an integral part of RJ. Moreover, there needs to be robust obligations in international and national frameworks to explicitly address intersectional needs in groups that experience disproportionate vulnerabilities and risks.

Finally, this study has implications beyond the specific case study of Venezuelan adolescent migrant girls in Colombia. It emphasizes the need to critically examine responses to SRHR for adolescent migrant girls in humanitarian settings (Ivanova et al. 2018; Mason-Jones and Nicholson 2018; Desrosiers et al. 2020). Responses should acknowledge the range of actors involved in delivering the response from private donors, governments, international organizations, service providers, and administrative staff. Policies should actively promote and prioritize adolescent-friendly, migrant-inclusive contraceptive education, information, and resources. This will result in responses that are better equipped to deal with the complex reproductive realities of adolescent migrant girls.

## Conclusion

Through the lens of RJ, this article argues that contraceptive care has been discouraged due to categorizing migrants as irregular, and framing contraception as “non-urgent.” Conceptualizing contraceptive care in this way means that instead of a continuum of SRH and contraceptive care, particular aspects of SRH are isolated and prioritized or deprioritized according to actors’ agendas. Here, the sole focus on emergency care has side-lined attempts to design longer-term, holistic responses that would provide primary care, education, and information.

International and national normative frameworks in the post-ICPD agendas have fallen short of advocating for comprehensive actions that simultaneously address the dimensions of age, gender, and migration—often treating contraceptive care, adolescent-friendly services, and humanitarian crises as separate silos of response. Here, responses should adopt the principles of RJ, which center intersectional reproductive realities. In this example, adolescent-friendly, migrant-inclusive contraceptive care needs to be available. While I remain hopeful, non-binding recommendations External Circular No. 35 of 2022, which promotes contraceptive care to adolescent migrant girls, are unlikely to have a significant impact given the uneven application of previous recommendations such as External Circular No. 25 of 2017. Further research is needed to assess the impact of these recommendations on service provision.

In practice, a short-term focus on “emergency care” in specific locations, for prescribed groups, working to meet predetermined indicators legitimizes ad hoc contraceptive care. Constraints are imposed by top-down donors, limiting the reproductive freedom of Venezuelan adolescent migrant girls. Thus, despite the efforts of the ICPD to eradicate such forms of population control, challenges persist. Furthermore, the failure to provide intersectional contraceptive care neglects to acknowledge that the realization of contraceptive care is reliant on addressing interconnected injustices that adolescent migrant girls face because of their age, gender, and migration status. Regarding the post-ICPD agenda, responses fail to address situations of vulnerability. To amend this, responses should avoid a piecemeal approach by adopting a holistic view of SRH as a continuum. Above all, responses should be intersectional to center the reproductive realities of adolescent migrant girls.

## Data Availability

Due to the sensitive nature of the questions asked in this study, interviewees were informed that anonymized data would be made available on request. The request form is available via the University’s data repository (Pure) 10.5258/SOTON/D3078.

## Appendix

**Appendix 1:** Major international and national normative frameworks for adolescent Venezuelan migrants'access to contraceptive care

Authority	Name	Year	Description
International Conference on Population and Development (ICPD)	ICPD Programme of Action	1994	Advocates for universal access to healthcare services, especially for family planning, and recognizes that migrants and displaced persons often have limited access to SRHR services. It emphasizes the importance of providing proper treatment, welfare services, and physical safety for documented migrants in compliance with international conventions and instruments
ICPD	ICPD Beyond 2014	2012	Names modern contraceptives as part of family planning. Importantly, it promoted a more holistic approach to adolescent SRHR that acknowledged the “intersecting forms of discrimination” including those faced by adolescent girls, as well as the persistent inequalities faced by migrants (UNFPA 2016)
ICPD	Nairobi Summit+25	2019	Sets the goals of zero unmet family planning needs and universal access to safe contraceptives; access for all adolescent girls, to “comprehensive and age-responsive information, education and adolescent-friendly services”; and finally, to uphold SRHR in humanitarian settings by ensuring that the “basic humanitarian needs and rights” of girls met through access to comprehensive SRH (UNFPA 2019a)
Inter-agency Working Group (IAWG) on Reproductive Health in Crises., UNFPA, Colombian Ministry of Health and Social Protection (MSPS)	The Practical Guide for the implementation of Minimum Initial Service Package in Colombia	2018	Identifies “minimum, life-saving” SRH needs that humanitarians must address at the onset of a crisis, in doing so it acknowledges adolescents differently affected. Addresses contraception as an “additional priority action.” It further recommends the provision of specific packages that address the basic supply of condoms and IUDs in humanitarian crises
MSPS	National Sexual and Reproductive Health Plan 2014–2021	2014	Focuses on three key objectives:1. Health promotion, through promoting SRR2. Health risk management through the management of factors affecting SRH3. Public health management through SRH actions
Government	External Circular No. 25	2017	Strengthening Public Health Actions to Respond to the Migration Situation of the Population from Venezuela guides the actions taken by territories to integrate Venezuelan migrants into the health system and guaranteed emergency healthcare to Venezuelan migrants regardless of their regularization or affiliation status
National Planning Department	COPNES 3950	2018	Requires the Ministry of Health to focus on immediate emergency response and basic needs such as temporary shelter, water, and sanitation at the border. SRHR policies were limited to emergency actions such as obstetric care
Government	Decree 216	2021	Adopts the Temporary Protection Statute for Venezuelan Migrants under the Temporary Protection Regime provides routes to grant regular status to anyone arriving irregularly before January 31, 2022, and for anyone entering regularly until May 28, 2023, for 10 years
Government	External Circular No. 35	2022	Recommendations for strengthening the inclusion and care of the Venezuelan migrant population in the General System of Social Security in Health. This emphasized integrating migrants into the general health system, including access to modern contraception information, education, and resources. Most importantly, it called for free access to contraceptives regardless of migrants'regularization status or affiliation to the health system

**Appendix 2:** Number of interviewees by sector and type of organization

Sector	Type of organization	Number of participants
Public health	State	3
Public health	Departmental	2
Health	Multilateral	4
Medical	Not-for-profit	4
Migration	Multilateral	3
Legal	NGO	3
Migration	State	2
Health (non-medical)	Foundation	3
Migration	NGO	1
Education	Medical	1
Education	NGO	1
Total number of key informants		27
Adolescent migrant girls from Venezuela in Bogota		3

*In total, 33 organizations were contacted. I discounted 7 because their services did not serve migrants or had no relation to SRHR. In total, there were 16 non state actors, 11 state actors, and 3 adolescent migrant girls, as seen above.

## Footnotes

1 Paragraph 7.11, Paragraph 7.3, Paragraph 7.45 of the 1994 ICPD Program of Action.

2 Non-state actors includes international organizations, non-governmental organizations, and civil societies. State actors include governmental departments and ministries.

3 I focus on older adolescents aged 15–19 years as defined by the WHO (2021).

4 Modern contraceptives include oral contraceptive pills, emergency contraception pills, implants, injectables, patches, vaginal rings, intrauterine devices, condoms, surgical methods, lactational amenorrhea method, standard days method, basal body temperature method, two-day method, and symptom-thermal method (WHO 2018).

5 Policy Statement of the United States of America at the United Nations International Conference on Population, 2 d Sess., Mexico City (Aug 6–13, 1984).

6 Correspondence with Ministry of Health and Social Protection, File No. 202216002491121, 14/12/2022.

7 A sub-cluster refers to a specialized working group or component within a larger humanitarian or emergency response cluster.

8 Author’s own experience in the field.

9 Principle 15 of the 1994 ICPD Program of Action.
